# Necrology

**Published:** 1902-09

**Authors:** 


### NECROLOGY.

JOHN FAUST, V. S. After a long illness, Dr. John Faust, one of the oldest and most highly esteemed veterinarians, passed away at his home in Poughkeepsie, N. Y. About ten years ago Dr. Faust received an injury to his spine as the result of a railroad accident, and his death was indirectly due to this cause. Several times it was thought he was on the road to recovery, but about a year ago he began to fail, and paralysis followed. He was confined to his bed about two months before his death.

The deceased was born in Hesse-Cassel, Germany, in 1835, and came to this country in 1852. He learned the cooper's trade, and in 1859 came to Poughkeepsie with his brothers, Tobias and Otto, and started in business under the name of John Faust & Son. In 1875 the partnership was dissolved, and Dr. Faust took up the study of veterinary surgery and passed his examination before the board of the New York Veterinary Society in 1881, when he received the degree of V. S.

Dr. Faust was a member of the American Veterinary Medical Association, and for several years served by appointment as cattle inspector for tuberculosis in this State. He received the credit of being the first to vaccinate against anthrax fever. He served with much credit as a member of the Board of Health for several years.

Joseph F. Lennon, M.D.C., of Joliet, Illinois, died on August 4th, at the age of thirty-six, from an operation for liver trouble of long standing. Dr. Lennon was a graduate of the Chicago Veterinary College, class of 1891, and had built up a large practice at Joliet.

Noah Cressy, M.D., Ph.D., D.V.S., died suddenly at his home in Hartford, Conn., August 31st, from kidney trouble. Dr. Cressy was sixty-three years old, a graduate of Pittsfield Medical College, and of the Montreal Veterinary College, class of 1878. At the time of death was editor of the *Connecticut Farmer*.

Dr. H. P. Kauffman, of Ashland, Pa., has removed to Shamokin, Pa., where he will continue the practice of his profession.

